# Magnetic resonance imaging‐based radiomics analysis for prediction of treatment response to neoadjuvant chemoradiotherapy and clinical outcome in patients with locally advanced rectal cancer: A large multicentric and validated study

**DOI:** 10.1002/mco2.609

**Published:** 2024-06-20

**Authors:** TingDan Hu, Jing Gong, YiQun Sun, MengLei Li, ChongPeng Cai, XinXiang Li, YanFen Cui, XiaoYan Zhang, Tong Tong

**Affiliations:** ^1^ Department of Radiology Fudan University Shanghai Cancer Center, Department of Oncology, Shanghai Medical College, Fudan University Shanghai China; ^2^ Department of Colorectal Surgery Fudan University Shanghai Cancer Center, Department of Oncology, Shanghai Medical College, Fudan University Shanghai China; ^3^ Department of Radiology Shanxi Province Cancer Hospital, Shanxi Hospital Affiliated to Cancer Hospital, Chinese Academy of Medical Sciences, Cancer Hospital Affiliated to Shanxi Medical University Taiyuan China; ^4^ Key Laboratory of Carcinogenesis and Translational Research (Ministry of Education/Beijing) Department of Radiology, Peking University Cancer Hospital and Institute Beijing China

**Keywords:** locally advanced rectal cancer; neoadjuvant chemoradiotherapy; treatment response; radiomics analysis

## Abstract

Our study investigated whether magnetic resonance imaging (MRI)‐based radiomics features could predict good response (GR) to neoadjuvant chemoradiotherapy (nCRT) and clinical outcome in patients with locally advanced rectal cancer (LARC). Radiomics features were extracted from the T2 weighted (T2W) and Apparent diffusion coefficient (ADC) images of 1070 LARC patients retrospectively and prospectively recruited from three hospitals. To create radiomic models for GR prediction, three classifications were utilized. The radiomic model with the best performance was integrated with important clinical MRI features to create the combined model. Finally, two clinical MRI features and ten radiomic features were chosen for GR prediction. The combined model, constructed with the tumor size, MR‐detected extramural venous invasion, and radiomic signature generated by Support Vector Machine (SVM), showed promising discrimination of GR, with area under the curves of 0.799 (95% CI, 0.760–0.838), 0.797 (95% CI, 0.733–0.860), 0.754 (95% CI, 0.678–0.829), and 0.727 (95% CI, 0.641–0.813) in the training and three validation datasets, respectively. Decision curve analysis verified the clinical usefulness. Furthermore, according to Kaplan–Meier curves, patients with a high likelihood of GR as determined by the combined model had better disease‐free survival than those with a low probability. This radiomics model was developed based on large‐sample size, multicenter datasets, and prospective validation with high radiomics quality score, and also had clinical utility.

## Graphical abstract

This study constructed a combined model with clinical MRI features and radiomics signature generated by Support Vector Machine (SVM) algorithm, and showed promising discrimination for the prediction of treatment response to neoadjuvant chemoradiotherapy (nCRT) in locally advanced rectal cancer (LARC) patients. The model was then validated in two external validation datasets and a prospective validation dataset, exhibited moderate performance for predicting good response, and was valuable for prognosis prediction.

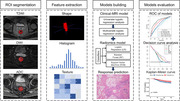

## 1 INTRODUCTION

Colorectal cancer is currently one of the most prevalent malignancies,^1^ with rectal cancer accounting for one‐third of these cases.^2^ The National Comprehensive Cancer Network guidelines currently recommend neoadjuvant chemoradiotherapy (nCRT) followed by total mesorectal excision (TME) and adjuvant chemotherapy as the standard treatment for locally advanced rectal cancer (LARC), which is defined as either clinical stage T3/4 or node‐positive disease, when R0 resection can be achieved.^3^

Approximately 15–27% of patients could achieve pathological complete response (pCR) after nCRT, in which all tumor cells completely vanish from surgical specimens.^4^ These people typically experience excellent long‐term results without TME, and a “wait and see” approach is advised to preserve a high standard of living.^5^, ^6^, ^7^, ^8^ For patients without pCR, some of them can also benefit from tumor downstaging, which could improve the resectability rate.^9^ However, some studies^9^, ^10^ reported that a small proportion of LARC patients still do not respond to nCRT. Therefore, it is of clinical significance to determine which patients may benefit from nCRT. However, the gold standard for tumor response evaluation is the histopathological examination of resected specimens. Early and noninvasive prediction of treatment efficacy and patient prognosis is an unsolved challenge.

Magnetic resonance imaging (MRI) can offer greater contrast‐to‐noise ratio and superior tissue contrast than other imaging modalities, and accurate evaluation of the depth of tumor infiltration, involvement of lymph nodes as well as mesorectal fascia (MRF) involvement and extramural venous invasion (EMVI) can be assessed on MRI.^11^, ^12^, ^13^ Previous studies have demonstrated that morphological features such as MR‐reported tumor size,^14^ T^15^ and N^16^ stage, and MR‐detected EMVI^15^, ^17^ may have the potential to identify treatment response to nCRT.

Unlike morphological features that are visible to the naked eye, radiomics, as a new image analysis technique, makes deep mining of medical images possible.^18^ Radiomics process has been proven to accurately predict tumor response,^19^ treatment benefit,^20^ and prognosis^21^, ^22^ in rectal cancer patients. In terms of the treatment response to nCRT, previous MRI‐based radiomics investigations have shown promising diagnostic performance. Liu et al.^23^ established an radiomics model using both pre‐ and post‐nCRT MR image data to predict pCR and achieved the highest area under the receiver operating characteristic (ROC) curve (area under the curve; AUC) of 0.9756 in the validation cohort, while other studies also performed well, with AUCs ranging from 0.75 to 0.948.^13^, ^24^, ^25^, ^26^, ^27^, ^28^, ^29^ However, most studies have focused on the prediction of pCR, and some of them had certain limitations, such as an insufficient number of patients for model training and validation,^24^, ^30^, ^31^ or a lack of external and prospective validation in multicenter institutions.^13^, ^29^

In light of these promising findings and in an effort to overcome the shortcomings of earlier research, we created the radiomics research based on large‐sample size and multicenter datasets. The aims of our study were (1) to develop and evaluate MRI‐based radiomics models using different classifiers to predict good response (GR) in LARC patients before nCRT and (2) to construct a combined model to select good responders with the clinical MRI features and radiomics signature generated by the best classifier, and further verify its value in prognosis prediction.

## 2 RESULTS

### 2.1 Patient baseline characteristics

**FIGURE 1:**
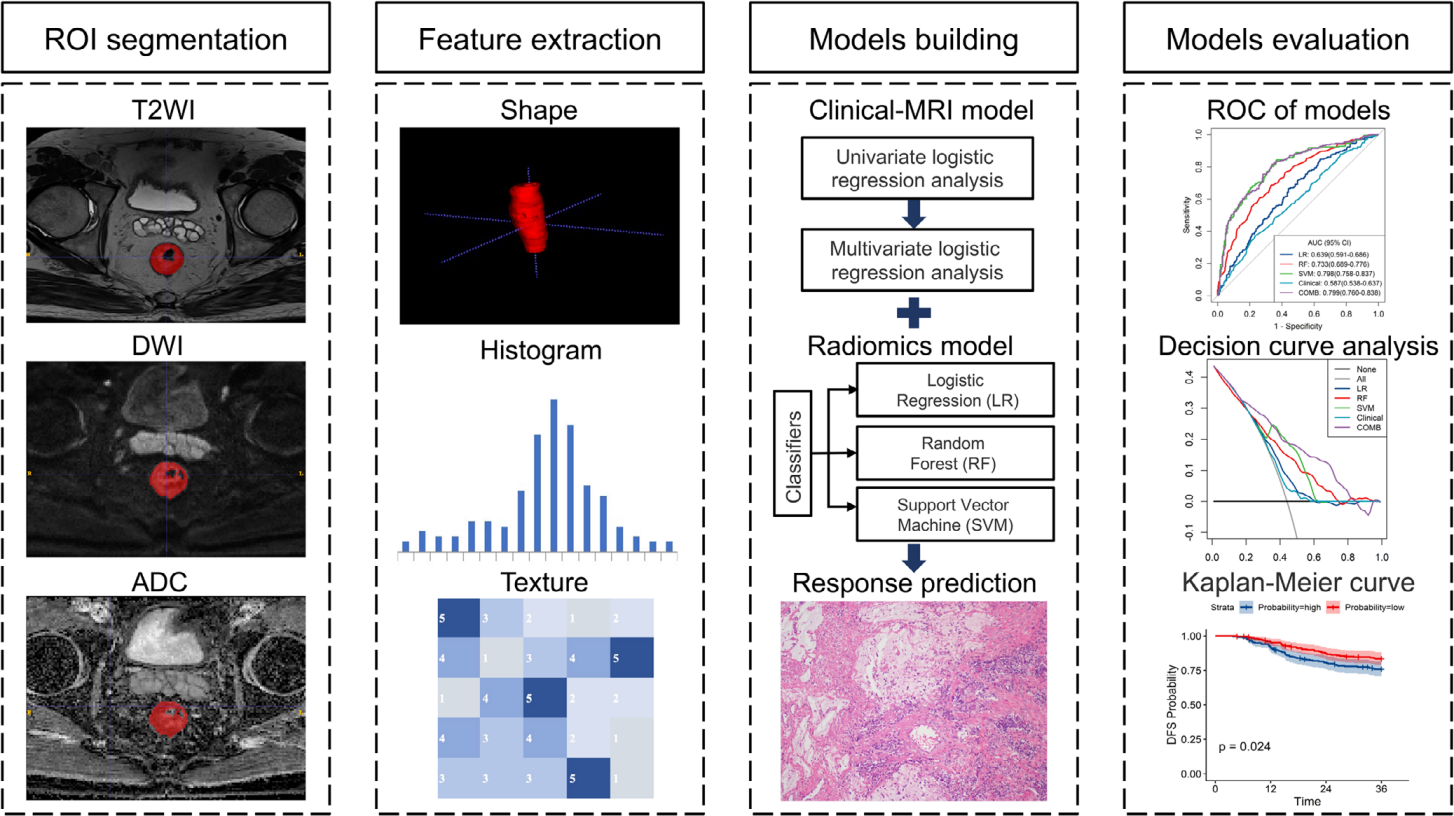
Workflow of this study.

A total of 1070 patients (359 females and 711 males; mean age 55.1 ± 10.9 years; range 21−79 years) who met the requirements for enrollment were included in this study, including 508 LARC patients retrospectively enrolled at Fudan University Shanghai Cancer Center (FUSCC) from May 2016 to January 2018 as the training dataset (TD); 242 LARC patients retrospectively enrolled at Beijing Cancer Hospital (BJCH) from December 2009 to May 2015 as the external validation dataset 1 (EVD1), 171 LARC patients retrospectively enrolled at Shanxi Province Cancer Hospital (SXPCH) from March 2013 to November 2016 as the external validation dataset 2 (EVD2), and 149 LARC patients prospectively enrolled from 2018/09 to 2023/08 derived from the clinical trials (Registration No. NCT03415763)^32^ at the FUSCC as the prospective validation dataset (PVD). Figure S1 shows the patients recruitment pathway. The GR rates were 44.1% (224 out of 508), 36.4% (88 out of 242), 46.2% (79 out of 171), and 57.0% (85 out of 149) for the TD, EVD1, EVD2, and PVD, respectively. Figure 1 shows the procedure of the study. A summary of the clinical and MRI characteristics of all individuals diagnosed with LARC are presented in Tables 1 and S1.

**TABLE 1:** Clinical and MRI features of patients with LARC.

	TD (*n* = 508)			EVD1 (*n* = 242)			EVD2 (*n* = 171)			PVD (*n* = 149)		
Features	GR (*n* = 224)	PR (*n* = 284)	*p* Value	GR (*n* = 88)	PR (*n* = 154)	*p* Value	GR (*n* = 79)	PR (*n* = 92)	*p* Value	GR (*n* = 85)	PR (*n* = 64)	*p* Value
Age (y)	55.5 (47.0–62.0)	57.0 (48.0–63.0)	0.130	55.4 (44.0–66.8)	54.8 (43.7–65.9)	0.650	53.8 (42.7–64.9)	54.9 (45.5–64.3)	0.493	57.0 (45.0–69.1)	57.5 (47.0–68.1)	0.784
Sex			0.262			0.186			0.258			0.632
Female	63 (28.1%)	93 (32.7%)		39 (44.3%)	55 (35.7%)		28 (35.4%)	39 (42.4%)		27 (31.8%)	18 (28.1%)	
Male	161 (71.9%)	191 (67.3%)		49 (55.7%)	99 (64.3%)		51 (64.6%)	53 (57.6%)		58 (68.2%)	46 (71.89%)	
CEA level			0.376			0.109			0.077			0.164
≤5 ng/mL	180 (80.4%)	219 (77.1%)		69 (78.4%)	106 (68.8%)		62 (78.5%)	61 (66.3%)		61 (71.8%)	39 (60.9%)	
>5 ng/mL	44 (19.6%)	65 (22.9%)		19 (21.6%)	48 (31.2%)		17 (21.5%)	31 (33.7%)		24 (28.2%)	25 (39.1%)	
Distance (cm)	5.0 (3.6–6.3)	5.5 (4.0–7.0)	0.076	4.9 (2.8–7.0)	5.3 (3.0–7.6)	0.157	5.0 (2.9–7.1)	5.9 (3.3–8.5)	0.018^*^	4.8 (2.9–6.6)	4.8 (2.8–6.8)	0.979
Tumor size (cm)	5.0 (4.0–6.0)	5.3 (4.3–6.3)	0.008^*^	5.1 (3.8–6.4)	5.7 (4.3–7.1)	0.001^*^	4.4 (3.3–5.5)	5.1 (3.8–6.4)	0.001^*^	5.0 (3.9–6.1)	5.3 (4.1–6.5)	0.075
mrT stage			0.044^*^			0.811			0.031^*^			0.093
T2	11 (4.9%)	8 (2.8%)		11 (12.5%)	19 (12.3%)		5 (6.3%)	6 (6.5%)		5 (5.9%)	1 (1.6%)	
T3	187 (83.5%)	222 (78.2%)		67 (76.1%)	113 (73.4%)		67 (84.8%)	64 (69.6%)		73 (85.9%)	51 (79.7%)	
T4	26 (11.6%)	54 (19.0%)		10 (11.4%)	22 (14.3%)		7 (8.9%)	22 (23.9%)		7 (8.2%)	12 (18.8%)	
mrN stage			0.095			0.068			0.101			0.949
N0	20 (8.9%)	19 (6.7%)		9 (10.2%)	5 (3.2%)		12 (15.2%)	6 (6.5%)		6 (7.1%)	4 (6.3%)	
N1	80 (35.7%)	81 (28.5%)		27 (30.7%)	57 (37.0%)		30 (51.9%)	31 (33.7%)		32 (37.6%)	23 (35.9%)	
N2	124 (55.4%)	184 (64.8%)		52 (59.1%)	92 (59.7%)		37 (32.9%)	55 (59.8%)		47 (55.3%)	37 (57.8%)	
mrMRF			0.034^*^			0.001^*^			0.323			0.217
Negative	156 (69.6%)	172 (60.6%)		67 (76.1%)	85 (55.2%)		53 (67.1%)	55 (59.8%)		60 (70.6%)	39 (60.9%)	
Positive	68 (30.4%)	112 (39.4%)		21 (23.9%)	69 (44.8%)		26 (32.9%)	37 (40.2%)		25 (29.4%)	25 (39.1%)	
mrEMVI			0.006^*^			0.018^*^			0.193			0.006^*^
Negative	152 (67.9%)	159 (56.0%)		51 (58.0%)	65 (42.2%)		58 (73.4%)	59 (64.1%)		60 (70.6%)	31 (48.4%)	
Positive	72 (32.1%)	125 (44.0%)		37 (42.0%)	89 (57.8%)		21 (26.6%)	33 (35.9%)		25 (29.4%)	33 (51.6%)	

Abbreviations: CEA, carcinoembryonic antigen; DFS, disease‐free survival.; EMVI, extramural venous invasion; EVD1, external validation dataset 1; EVD2, external validation dataset 2; GR, good response; MRF, mesorectal fascia; PR, poor response; PVD, prospective validation dataset; TD, training dataset.

* *p* < 0.05.

In the TD, EVD1, and EVD2, patients with bigger tumor sizes were more likely to achieve poor response (PR) (*p* < 0.05). In the TD and EVD1, the GR group exhibited less involvement of mrMRF (TD: 30.4 vs. 39.4%, *p* = 0.034; EVD1: 23.9 vs. 44.8%, *p* < 0.001) and mrEMVI (TD: 32.1 vs. 44.1%, *p* = 0.006; EVD1: 42.0 vs. 57.8%, *p* = 0.018) than the PR group. In the TD and EVD2, more advanced mrT stages were detected in the PR group. In the EVD2, patients who achieved GR exhibited a shorter distance from the anus than did those in the PR group (5.0 ± 2.1 vs. 5.9 ± 2.6 cm, *p* = 0.018). In the PVD, only positive mrEMVI was found to be more common in the PR group (51.6 vs. 29.4%, *p* = 0.006), while no significant differences were observed in other clinical or MRI features between the PR and GR groups. Furthermore, no statistically significant differences were observed between the PR and GR groups in any of the four datasets concerning age, sex, carcinoembryonic antigen (CEA) level, or mrN stage (*p* > 0.05).

### 2.2 Clinical‐MRI model performance

In the TD, univariate Cox regression analysis revealed that greater distance from the anus, bigger tumor size, more advanced mrT and mrN stage, and greater mrMRF and mrEMVI involvement were significantly associated with PR. No significant differences were observed in terms of sex, age, or CEA level for GR prediction. Stepwise multivariate analysis revealed that a small tumor size (hazard ratio [HR] = 0.888, [95% confidence interval [CI] 0.795–0.992]; *p* = 0.036) and a negative mrEMVI (HR = 0.655, [95% CI 0.450–0.951]; *p* = 0.026) were independently predictive of a higher probability of GR. Ultimately, utilizing the size and mrEMVI (Table 2), a clinical‐MRI model was constructed to predict GR (Table 2). The TD showed an AUC of 0.587 (95% CI, 0.538–0.637). This was later verified with AUCs of 0.635 (95% CI, 0.563–0.708) in the EVD1, 0.636 (95% CI, 0. 552–0.719) in the EVD2, and 0.643 (95% CI, 0.553–0.732) in the PVD, respectively.

**TABLE 2:** Logistic regression analysis for GR prediction in the training dataset.

Characteristic	Univariate logistic regression		Multivariate logistic regression	
Variables and intercept	OR (95%CI)	*p*	OR(95%CI)	*p*
Sex (male vs. female)	1.244 (0.849–1.824)	0.263	NA	NA
Age (years)	0.988(0.972–1.004)	0.137	NA	NA
CEA level (0–5 vs. > 5 ng/mL)	0.824(0.536–1.267)	0.377	NA	NA
Distance (per 1 mm increase)	0.916 (0.841–0.998)	0.046^*^	NA	NA
Tumor size (per 1 mm increase)	0.866 (0.777–0.965)	0.009^*^	0.888 (0.795–0.992)	0.036^*^
mrT stage (T2 vs. T3 vs. T4)	0.581 (0.378–0.893)	0.013^*^	NA	NA
mrN stage (N0 vs. N1 vs. N2)	0.749 (0.568–0.987)	0.040^*^	NA	NA
MRF((−) vs. (+))	0.669 (0.462–0.970)	0.034^*^	NA	NA
EMVI((−) vs. (+))	0.603 (0.418–0.868)	0.007^*^	0.655 (0.450–0.951)	0.026^*^

Abbreviations: CEA, carcinoembryonic antigen; CI, confidence interval; EMVI, extramural venous invasion; GR, good response; MRF, mesorectal fascia; OR, odds ratio.

* *p* < 0.05.

### 2.3 Construction and validation of radiomics models

Based on inter‐ and intra‐observer evaluations, 1200 of the 1237 radiomics features showed an inter‐/intra‐class correlation coefficient (ICC) > 0.75. The radiomics features with an ICC < 0.75 (26 features extracted from T2 weighted (T2W) images and 11 features extracted from Apparent diffusion coefficient (ADC) images) are listed in Table S2. Of these, 857 features (806 from T2W images and 51 from ADC images) were retained after the Mann‒Whitney *U* test, and 20 features (nine from T2W images and 11 from ADC images) were retained after Spearman's correlation analysis. Finally, 10 radiomics features, including eight from T2W images (log.sigma.3.0.mm.3D_glszm_ZoneEntropy, log.sigma.3.0.mm.3D_firstorder_Minimum, Wavelet.LLL_glcm_DifferenceAverage, Wavelet.LHH_glcm_MaximumProbability, Wavelet.LHH_firstorder_Minimum, Wavelet.LLL_glszm_LowGrayLevelZoneEmphasis. Wavelet.LLH_glcm_Idn, Wavelet.LHH_glszm_SmallAreaLowGrayLevelEmphasis) and two from ADC images (original_gldm_GrayLevelNonUniformity, original_gldm_DependenceEntropy), were selected by the Boruta feature selection method to establish the radiomics models.

**FIGURE 2:**
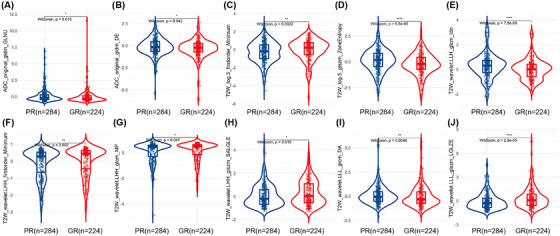
Plots (A–J) show the boxplots of the ten radiomics features with significant differences between the GR and PR groups in the training dataset. GR, good response; PR, poor response.

The 10 features mentioned above, all of which demonstrated significant differences between the PR and GR groups (all *p* values < 0.05), were used to construct further radiomics models after the feature selection process (Figure 2). The importance of these features is displayed in Figure S2. The correlation matrix of the selected features is showed in Figure S3. Based on the logistic regression (LR), random forest (RF), and support vector machine (SVM) algorithms, radiomics models with selected features were built.

**FIGURE 3:**
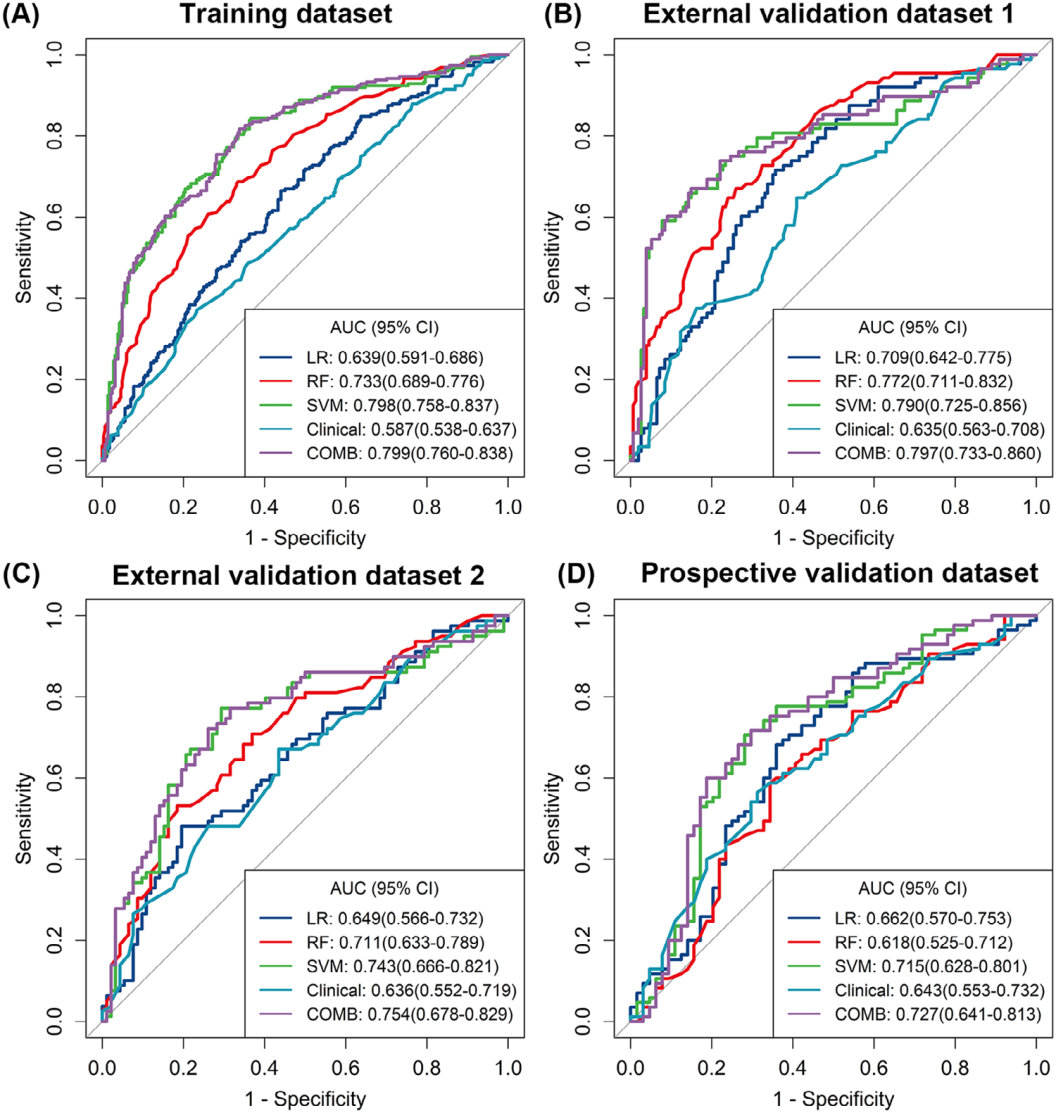
Receiver operating characteristic curves of the five models for predicting good response. AUC, area under the curve; CI, confidence interval;COMB, the combined model; LR, logistic regression; RF, random forest; SVM, support vector machine.

The predictive performance is displayed in Table 3, with AUCs ranging from 0.639 to 0.798 in the TD. With an AUC of 0.798 (95% CI, 0.758–0.837) in the TD and a cutoff value of 0.372, the radiomics model derived from the SVM performed best for predicting GR. The AUCs for EVD1, EVD2, and PVD were 0.790 (95% CI, 0.725–0.856), 0.743 (95% CI, 0.666–0.821), and 0.715 (95% CI, 0.628–0.801), respectively (Figure 3).

**TABLE 3:** Diagnostic performance of different models.

	Radiomics models based on LR classifier	Radiomics models based on RF classifier	Radiomics models based on SVM classifier	Clinical‐MRI model	Combined model
TD (*n* = 508)					
AUC (95%CI)	0.639 (0.591–0.686)	0.733 (0.689–0.776)	0.798 (0.758–0.837)	0.587 (0.538–0.637)	0.799 (0.760–0.838)
ACC (95%CI)	0.606 (0.562–0.649)	0.675 (0.633–0.716)	0.726 (0.685–0.765)	0.593 (0.548–0.636)	0.730 (0.689–0.768)
Sensitivity	0.665 (0.545–0.728)	0.688 (0.585–0.752)	0.844 (0.745–0.880)	0.371 (0.277–0.437)	0.817 (0.723–0.857)
Specificity	0.560 (0.454–0.609)	0.665 (0.559–0.750)	0.634 (0.482–0.687)	0.768 (0.670–0.832)	0.662 (0.546–0.732)
EVD1 (*n* = 242)					
AUC (95%CI)	0.709 (0.642–0.775)	0.772 (0.711–0.832)	0.790 (0.725–0.856)	0.635 (0.563–0.708)	0.797 (0.733–0.860)
ACC (95%CI)	0.653 (0.589–0.713)	0.690 (0.628–0.748)	0.736 (0.675–0.790)	0.665 (0.602–0.724)	0.740 (0.680–0.794)
Sensitivity	0.727 (0.602–0.852)	0.705 (0.595–0.841)	0.773 (0.659–0.864)	0.330 (0.204–0.455)	0.750 (0.648–0.830)
Specificity	0.610 (0.493–0.721)	0.682 (0.565–0.808)	0.714 (0.331–0.818)	0.857 (0.707–0.925)	0.734 (0.519–0.870)
EVD2 (*n* = 171)					
AUC (95%CI)	0.649 (0.566–0.732)	0.711 (0.633–0.789)	0.743 (0.666–0.821)	0.636 (0.552–0.719)	0.754 (0.678–0.829)
ACC (95%CI)	0.596 (0.519–0.671)	0.667 (0.591–0.737)	0.673 (0.597–0.742)	0.579 (0.501–0.654)	0.678 (0.603–0.748)
Sensitivity	0.684 (0.532–0.797)	0.684 (0.504–0.797)	0.797 (0.709–0.911)	0.481 (0.392–0.658)	0.785 (0.709–0.899)
Specificity	0.522 (0.359–0.652)	0.652 (0.485–0.772)	0.565 (0.239–0.772)	0.663 (0.553–0.848)	0.587 (0.326–0.804)
PVD (*n* = 149)					
AUC (95%CI)	0.662 (0.570–0.753)	0.618 (0.525–0.712)	0.715 (0.628–0.801)	0.643 (0.553–0.732)	0.727 (0.641–0.813)
ACC (95%CI)	0.651 (0.569–0.727)	0.617 (0.534–0.696)	0.664 (0.583–0.740)	0.577 (0.494–0.658)	0.698 (0.617–0.770)
Sensitivity	0.706 (0.529–0.859)	0.635 (0.435–0.776)	0.788 (0.706–0.894)	0.435 (0.259–0.647)	0.765 (0.659–0.871)
Specificity	0.578 (0.438–0.750)	0.594 (0.390–0.750)	0.500 (0.281–0.750)	0.766 (0.652–0.875)	0.609 (0.390–0.781)

Abbreviations: ACC, accuracy; AUC, area under the curve; CI, confidence interval; EVD1, external validation dataset 1; EVD2, external validation dataset 2; LR, logistic regression; PVD, prospective validation dataset; RF, random forest; SVM, support vector machine; TD, training dataset.

### 2.4 Performance of the combined model for GR prediction and prognostic evaluation

**FIGURE 4:**
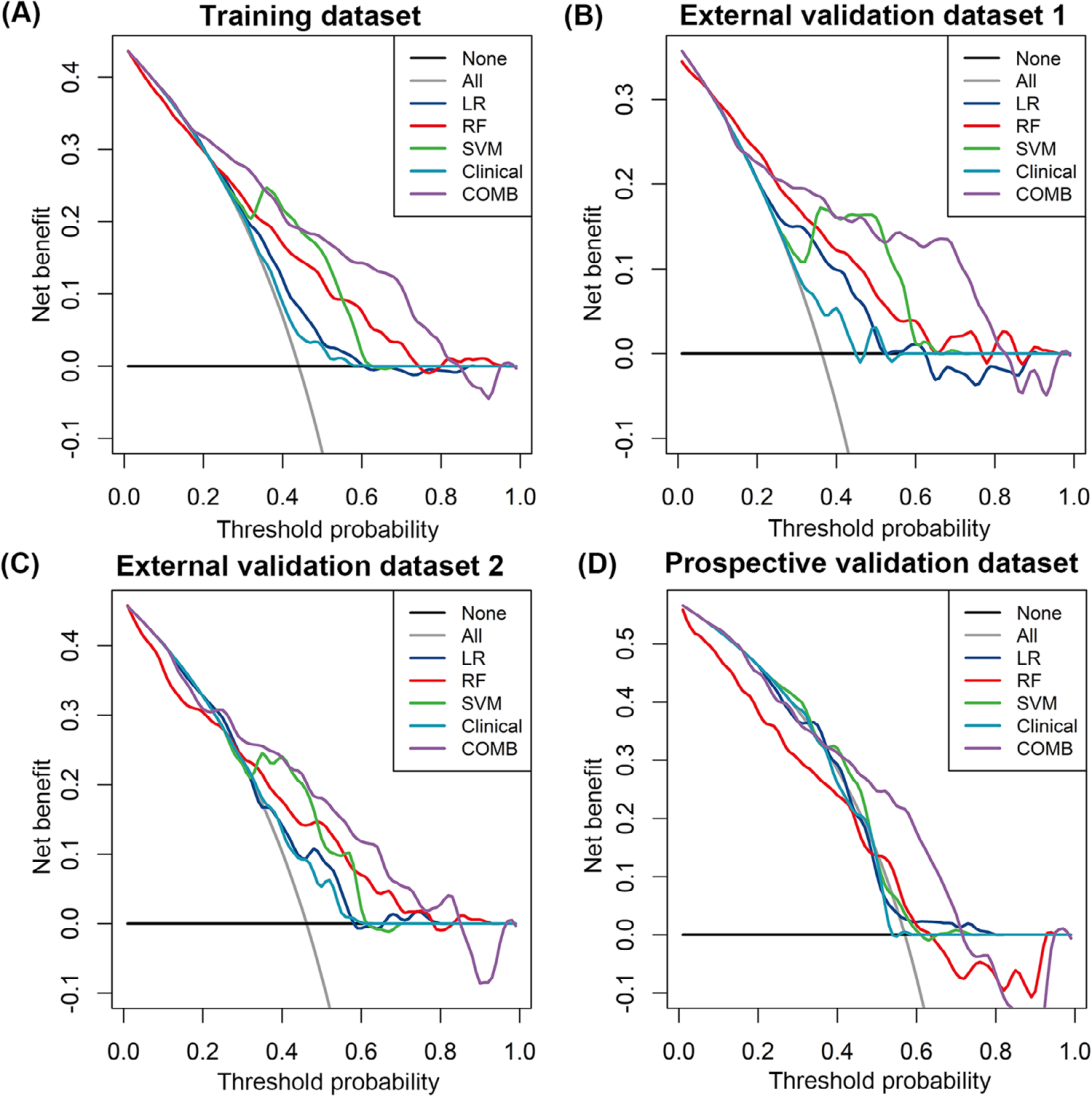
Decision curve analysis for predicting good response with the five model models. COMB, the combined model; LR, logistic regression; RF, random forest; SVM, support vector machine.

The combined model was constructed with the tumor size, MR‐detected EMVI and radiomics signature generated by the SVM algorithm. The AUC of the combined model for the prediction of GR was 0.799 (95% CI, 0.760–0.838; accuracy 0.730; sensitivity 81.7%; specificity 66.2%) in the TD, 0.797 (95% CI, 0.733–0.860; accuracy 0.740; sensitivity 75.0%; specificity 73.4%) in the EVD1, 0.754 (95% CI, 0.678–0.829; accuracy 0.678; sensitivity 78.5%; specificity 58.7%) in the EVD2, and 0.727 (95% CI, 0.641–0.813; accuracy 0.698; sensitivity 76.5%; specificity 60.9%) in the PVD with a cutoff value of 0.328, which was slightly greater than that of the SVM model (*p* = 0.775). Additionally, the decision curve analysis (DCA) revealed that the combined model provided a higher net benefit within a certain threshold range (Figure 4). The radiomics quality score (RQS) of the model was 27 out of 36 (Table S3).

**FIGURE 5:**
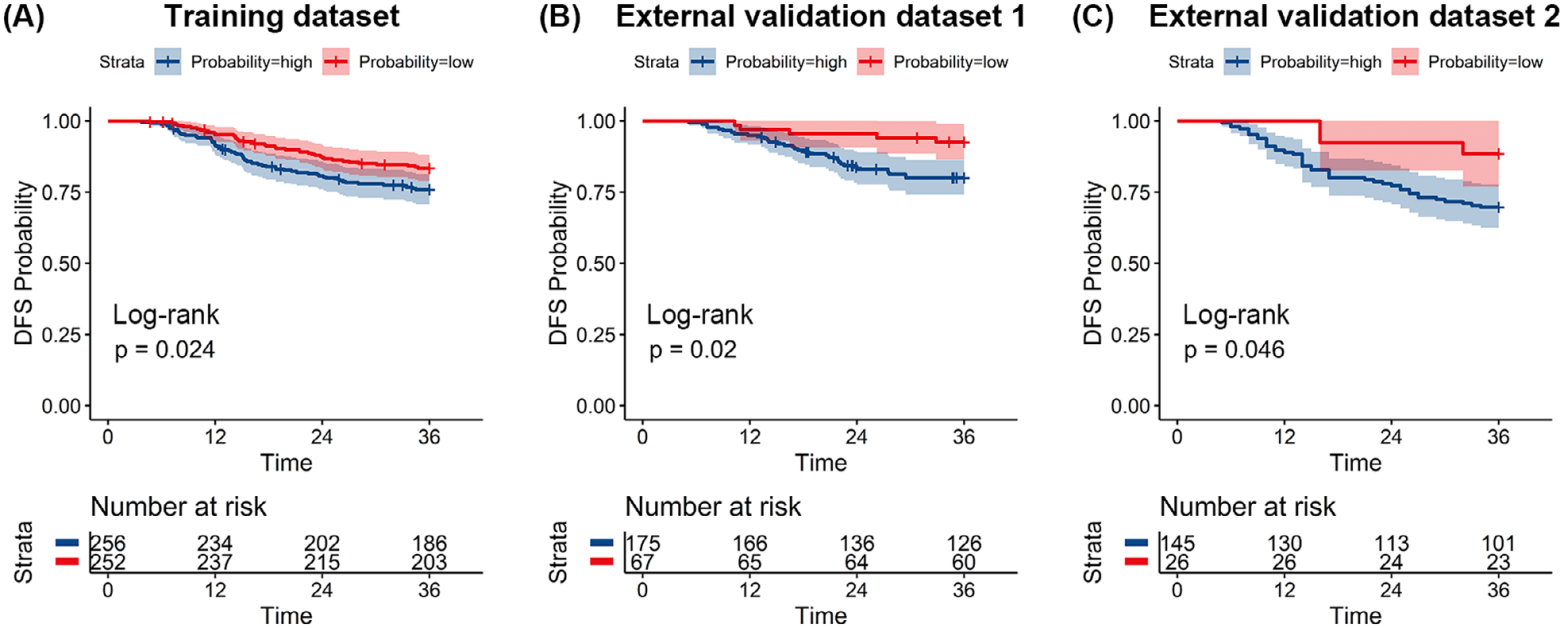
Kaplan–Meier curves of disease‐free survival (DFS) between the groups with high probability and low probability of having a good response defined by the combined model.

The median follow‐up periods in the TD, EVD1, and EVD2 were 55 (range, 6−116) months, 76.5 (range, 13−135) months, and 46 (range, 6−80) months, respectively. The Kaplan–Meier survival curves in all three datasets showed a significant difference in disease‐free survival (DFS) between the true GR and PR groups (all *p* < 0.05) (Figure S4). We evaluated the prognostic value of the combined model in the three datasets. Figure 5 shows the Kaplan–Meier survival analysis of the three datasets, and patients with a high probability of having a GR, as predicted by the combined model, had longer DFS than those with a low probability in all datasets (*p* < 0.05).

## 3 DISCUSSION

Previous studies have demonstrated the value of radiomics in predicting the response of LARC patients. Our research verified that the MRI‐based radiomics models outperformed the clinical‐MRI model and can accurately predict responses. In this multicenter study, the combined model obtained by SVM algorithms and clinical‐MRI features had the best performance with respect to discriminating LARC patients who are likely to be good responders to nCRT, yielding higher AUCs than did the clinical‐MRI model and the radiomics models based on RF, LR, and SVM classifiers in the TD and three validation datasets, and providing a complementary pathway of prognosis in patients with LARC.

There is a lack of extensive validation of an MRI‐based approach to assess great response in individuals with LARC following CRT. Compared with other radiomics studies in predicting a GR in LARC patients,^33^, ^34^, ^35^, ^36^, ^37^, ^38^, ^39^ our study has three major strengths, including the selection of routine examination sequences, the large sample size and prospective validation.

First, for the applicability of the model, we used MR images for radiomics analysis, as pelvic MRI, rather than CT^40^ or (18)F‐FDG PET/CT,^31^ is currently the most commonly used examination for staging newly diagnosed rectal cancer. Radiomics studies based on T2W images, ADC/DWI images and contrast‐enhanced T1W images achieved remarkable predictive efficacy, with an AUC of 0.944 in the validation cohort,^26^ although with the slightly different task of identifying patients who achieved pCR, our study only included T2W and ADC images. Given that contrast T1W did not improve diagnostic accuracy relative to high‐resolution small field of view T2WI with respect to assessments of T‐stage or MRF involvement^41^ and was not was not recommended as a routine sequence by either the European Society of Gastrointestinal and Abdominal Radiology or the Society of Abdominal Radiology at initial phase,^42^, ^43^ while a T2W sequence is mandatory^3^ and a DWI sequence is recommended,^42^ contrast T1W sequence was not analyzed in our study, leading to our model having a slightly lower predictive efficacy than others.^33^ However, the performance of the model based on multiple sequences could improve its predictive efficacy compares with that of a single‐sequence model^26^ because there is complementary information in different MRI sequences, which suggests that the value of using T1W sequence is worthy of investigation.

The second strength is the large sample size of more than 1000 patients for the model training and validation, especially the use of three independent datasets for external validation. A multicenter study can not only increase the robustness of the findings and reduce selection bias but also better address the reproducibility and reliability across populations; however, this type of study also leads to the inevitable problem of having a range of MR scanners and diverse imaging parameters from different manufacturers not only among various facilities but also within the same organization. To minimize the impact of acquisition technical factors on the reliability of the acquisition of radiomics features, we employed the *z*‐score method to normalize each feature by removing the mean and scaling to unit variance. This replicates real‐life practice and therefore dramatically increases the generalizability of the study findings compared with other studies using a single device with homogenous settings.^26^

Third, we proposed prospective validation in a clinical trial cohort. We noticed significant differences in the distribution of treatment responses between datasets, particularly in the prospective cohort. As reported by the CAO/ARO/AIO‐12 study^44^ nCRT followed by chemotherapy before TME resulted in a greater pCR. A similar trend was also observed in the PVD, in which a GR rate of 57.0% (85 out of 149) was achieved due to the three cycles of consolidation chemotherapy follow by nCRT and was higher than that in the other three datasets. However, the SVM‐based radiomics model also exhibited moderated efficacy for predicting GR in the PVD. Song et al.^45^ reported that there was no discernible improvement in the discriminatory power of a prediction model that integrated neoadjuvant treatment modalities. A possible explanation may be that the intrinsic heterogeneity of pretreatment tumors may have a greater impact on treatment resistance than that of neoadjuvant treatment regimens; thus, the radiomics model based on images at the initial phase was still able to predict the treatment response to slightly differences in nCRT regimens. Furthermore, due to variations in individual patient profiles and physician preference choices, the number of chemotherapy cycles for consolidation therapy and radiotherapy modalities varied among different studies, in contrast to clinical trials with a high degree of treatment therapy uniformity. Additionally, the enrolled population was not equally targeted in real clinical practice, which may have resulted in variations in the distribution of GR in different populations. With this in mind, our model still achieved good predictive efficacy in cohorts where differences existed, highlighting the stability and repeatability of the model.

Different classifiers affect the model performance; however, there is no consensus on the optimal choice of classifiers. In our study, the SVM model outperformed the RF and LR models in treatment response prediction. The SVM algorithm is particularly good at identifying subtle patterns in complex datasets because of its capacity to minimize classification errors on unseen data without making any assumptions about the probability distribution of the data beforehand. This makes the algorithm advantageous in modeling moderate nonlinearities, given the complexity and nonlinearity between radiomics and tumor response.^46^, ^47^ In other rectal cancer‐related studies, the SVM model also achieved the best predictive efficacy, such as in the prediction of KRAS mutations^48^ and pathological features^49^; however, there are also reports related to the superiority of the RF^30^, ^50^, ^51^, ^52^ and Bayes^53^ classifiers over SVM models in predicting different endpoints. Therefore, the best classifier may vary in different clinical applications, and no classifier is better than any other for all problems. Exploring the optimal classifier for different clinical application scenarios will be necessary and encouraged to in future studies.

We also evaluated the prognostic value of the radiomics model. There are few articles on the stratification of the prognoses of LARC patients after nCRT using the probability of GR as predicted by a radiomic model. Wang et al.^39^ constructed a radiomics model to predict treatment response in LARC patients, and the radiomics‐based nomogram showed separation of survival curves according to progression‐free survival. In our study, the combined model defined the high‐ and low‐risk probability groups for GR prediction, and the results showed a significant difference in survival outcomes: patients with a high probability of having a GR had a significantly longer DFS than those with a low probability of having a GR. As a result, the classification of patients into high‐ and low‐risk groups with varying DFS may be aided by our suggested radiomics model.

Our study has several limitations. First, because the reference standard for our analysis was based on histopathological findings, we may have introduced selection bias by excluding patients who had a clinical complete response under watch‐and‐wait therapy. Second, because DWI sequences are prone to artefacts and *b* values may differ among institutions, radiomics features extraction was not performed on DWI images, and ADC maps were analyzed instead. Of note, the regions of interest (ROI) did not include lymph nodes, which is a certain limitation, as residual nodal disease is important in clinical decision making. Third, manual segmentation of ROIs is a time‐consuming procedure and requires accurate identification of MR lesions, which can be challenging for clinicians lacking the experience in reading MR images. Thus, it is necessary to develop an automated or semiautomated tool to optimize the radiomics procedures. Finally, deep learning (DL) technology characterized by a convolutional artificial neural network have proven to be exceptionally effective in the fields of tumor subtype recognition,^54^ diagnosis,^55^ prognosis,^21^, ^56^ and treatment prediction.^57^ The DL technique has shown the advantages of segmentation, registration, and classification over supervised machine learning algorithms.^58^ Therefore, more study should be done on the application of DL algorithms in conjunction with MR‐based radiomics to predict the treatment response of LARC patients.

In conclusion, based on large sample size, routinely imaging sequences, multicenter datasets and prospective validation, our study constructed a robust, pervasive, and generalizable radiomics model for GR prediction and may also provide value in prognostic risk stratification. This study was conducted in accordance with the criteria of RQS and was a high‐quality radiomics investigation with a valuable predictive model worthy of clinical utility.

## 4 MATERIAL AND METHODS

### 4.1 Patients

This multicenter study involved a retrospective cohort for model development, two retrospective cohorts and one prospective cohort for model validation. Patients were eligible if they fulfilled the following inclusion requirements: (a) pathologically confirmed primary rectal adenocarcinoma by endoscopic biopsy; (b) nonmetastatic disease; (c) underwent baseline MRI before any treatment; (d) long‐term neoadjuvant therapy that combines capecitabine‐based chemotherapy and radiation (total dosage 45−50 Grey); (d) underwent TME within 12 weeks of complete nCRT treatment and achieved complete (R0) resection; (f) sufficient clinicopathologic characteristics; and (g) available and detailed histopathological results derived from the postoperative specimens. The following were the exclusion criteria: (a) poor image quality with significant artifacts or primary tumors that are not recognized on MR images; (b) evidence of distant metastasis or other primary malignant tumors; and (c) previous anticancer therapy before baseline MRI.

All patients received nCRT before TME. The nCRT regimen included radiation and concurrent chemotherapy. Pelvic radiation therapy was administered to the patients at a dose of 45−50 Gy/25–28 fractions, along with 825 mg/m^2^ of concurrent capecitabine twice daily for 5 days/week. TME was performed at least 4 weeks after the completion of nCRT. The completion of nCRT and TME occurred at a median interval of 8 weeks (range: 4−12 weeks).

This multicenter study was conducted in accordance with the Declaration of Helsinki. For studying patients in the TD, EVD1, and EVD2, ethics approvals were obtained from the Institutional Review Board of FUSCC (Shanghai, China; Approval No.1612167−18), BJCH (Beijing, China; Approval No.2020KT53), and SXPCH (Shanxi, China; Approval No.202011), respectively. The study's retrospective design allowed for the waiver of the informed consent requirement. For studying patients in the PVD, the study was approved by the Ethics Committee of FUSCC (Shanghai, China; Approval No.1807188−10). Informed consent was obtained from each patient, and written consent was obtained from the study participants.

### 4.2 Assessment of clinical and MRI features

Clinical features was obtained from medical records, including patient age, sex, and CEA level before nCRT. MRI morphological features, including the distance of the tumor from the anus (measured on T2 sagittal images), the size of the tumor (measured on T2 sagittal images), mrT stage, mrN stage, and MR‐detected MRF and EMVI were independently evaluated by two radiologists (reader 1 and reader 2, both having more than 5 years of rectal MRI experience). In cases of disagreement, a third radiologist (reader 3, with 20 years of experience in rectal MRI) was consulted, and the majority value was used. The clinical results and postoperative pathology findings were concealed from the three radiologists. The evaluation criteria for MRI morphological features and typical MR images of MRF and EMVI (Figure S5) can be obtained from Supporting Information.

### 4.3 MRI acquisition and tumor segmentation

The MRI acquisition protocol is illustrated in Supporting Information, and the detailed MRI parameters utilized at the three hospitals are shown in Table S4. Using ITK‐SNAP software (version: 3.4.3, www.itksnap.org), a colorectal MRI radiologist (reader 1) manually drew the ROIs along the tumor border on each successive slice of the entire tumor on T2WI and DWI (with a *b* value of 800 s/mm^2^ and then mapped to the ADC images). The ROI was outlined on each consecutive slice of the whole tumor volume without the surrounding lymph nodes, and the cystic and necrotic areas were manually excluded. Anatomical information from T2W images was carefully included for reference during the segmentation of DWI.

### 4.4 Reproducibility of radiomic feature extraction

In order to evaluate the inter‐ and intra‐observer reproducibility, a total of fifty patients were chosen at random, and their MRI scans were separately delineated again a month later by reader 1 and reader 3. Both readers were blinded to the pathological findings and clinical outcomes. The ICCs were calculated. Generally, both inter‐/intra‐observer ICCs ≥ 0.75 are regarded as indicating in good agreement.

### 4.5 Response assessment

Two gastrointestinal pathologists assessed the surgical resection specimens. Tumor regression grade (TRG) system of the 2010 American Joint Committee on Cancer was used to evaluate the pathological tumor response.^59^ The details of the TRG system were defined as follows: no viable cancer cells are classified as TRG 0; single or small groups of tumor cells are classified as TRG 1; residual cancer outgrown by fibrosis but with fibrosis still predominating are classified as TRG 2; and minimal or no tumor cells eliminated are classified as TRG 3. The patients were then split into two different response groups: the PR group (TRG 2−3) and the GR group (TRG 0−1).

### 4.6 Follow‐up and clinical endpoints

During the first 2–3 years, the patients were routinely followed up every 3–6 months, and then every 6–12 months after that. The minimum follow‐up period for patients without distant metastasis/recurrence was 36 months after surgery in this study. The endpoint of patients in the TD, EVD1, and EVD2 was metastasis/recurrence. Metastasis to an organ or region outside the pelvis was defined as distant metastasis. Recurrence was assessed in terms of either local or regional recurrence. Using histology or imaging, every case of distant metastasis or recurrence was verified. DFS was determined at the earliest instance of either tumor‐related death or recurrent disease (local, distant, or regional). The last follow‐up time was December 2021. Survival analyses were not performed in the PVD because the vast majority of patients did not meet the minimum follow‐up‐time requirement of 36 months.

### 4.7 Radiomics features extraction and selection

Using the free and open‐source PyRadiomics program (http://PyRadiomics.readthedocs.io/en/latest/), image preprocessing and feature extraction were carried out. Since the MRI images were collected from multiple centers, a series of image standardization techniques were applied to process the T2W and ADC images. The intensity of the T2WI image was normalized by centering it at the mean with *z*‐score method and a scale of 100. Then, we used a cubic B‐spline image interpolation algorithm to resample the T2WI and ADC images with resolutions of [1 × 1 × 1 mm^3^] and [1.5 × 1.5 × 1.5 mm^3^], respectively. The grey level of T2W images was quantized to 5 grey levels. The grey level of the ADC images was quantized to 15 grey levels. Radiomics features were extracted from the 3D segmentation of the tumor in all four datasets. Supporting Information presented the details of the feature extraction algorithms.

We used the data of the TD to conduct the feature selection process. Our method for choosing robust radiomics features with ICCs ≥ 0.75 consists of three steps. First, GR‐related features (with *p* < 0.05) were selected using the Mann–Whitney *U*‐test. Second, the redundant information was removed using Spearman's correlation analysis with *r* ≥ 0.90. If two variables had a high correlation, the variable with the largest mean absolute correlation was removed. Third, Boruta feature selection^60^ was performed to retain the final radiomics features (Supporting Information). Then, the selected robust radiomics features were used for further model building.

### 4.8 Model evaluation and survival analysis

We constructed five models, including a clinical‐MRI model, three radiomics models and a combined model. The relationship between the clinical‐MRI features and GR status was evaluated using univariate LR analysis, and the clinical‐MRI model was developed by taking into account the relevant features in multivariate LR analysis. By using the optimal subset of 10 robust radiomics features, we constructed three radiomics prediction models based on different classifiers, including LR, RF and SVM classifiers. The radial basis function was utilized as the kernel function for the SVM classifiers. The model with the greatest performance was chosen through training with 10 times fivefold cross‐validation; the optimal parameters were *C* = 3.280 and sigma = 0.084. The radiomics signature generated by the best classifier was defined as a new feature set and further used to construct a combined model with independent clinical‐MRI features by using the multivariate LR analysis. All models were constructed with the features of the TD and then applied to the three validation datasets.

Afterwards, the prediction abilities of the five models was then evaluated in the validation datasets. The ROC curves were plotted and the best cutoff point was obtained by maximizing the Youden index in the TD and applied in three validation datasets. To determine if there was a statistically significant difference between two randomly chosen ROC curves, the Delong test was applied. The AUC was calculated, and the sensitivity, specificity, and predictive accuracy were measured. The 95% CI of the AUC was determined by the bootstrap resampling method with 1000 replicates. DCA was performed to estimate the clinical usefulness of the models at different threshold probabilities.

We also investigated whether patients with different probabilities of GR predicted by the combined model could be stratified according to different risks of metastasis/recurrence in these datasets. Based on the optimum cutoff values identified by using the maximally selected rank statistical method of the combined model, the patients were categorized into low‐ and high‐probability GR groups. Kaplan–Meier survival analysis was performed and the Log‐rank test was used to compare DFS between the low‐ and high‐probability GR groups in the TD, EVD1, and EVD2.

### 4.9 Statistical analysis

We used SPSS software (version 21) and R software (version 4.2.1, www.R‐project.org) to perform all the statistical analyses. The differences in the clinical MRI features between the patients in different groups or datasets were compared using Fisher's exact test or the Chi‐squared test for categorical variables and the independent *t*‐test or the Mann‒Whitney *U*‐test for continuous variables, as appropriate. A two‐sided *p* < 0.05 was considered statistically significant. The RQS developed based on the expert opinions of Lambin et al.^18^ was used to guarantee a high standards of reporting and scientific rigor in radiomics studies.

## AUTHOR CONTRIBUTIONS

*Conceptualization*: TingDan Hu and Tong Tong. *Data curation*: TingDan Hu, Menglei Li, and ChongPeng Cai. *Formal analysis*: TingDan Hu. *Funding acquisition*: Tong Tong and Yiqun Sun. *Investigation*: TingDan Hu, Jing Gong, and Yiqun Sun. *Methodology*: TingDan Hu and Jing Gong. *Project administration*: Tong Tong, Xiaoyan Zhang, Yanfen Cui, and Xinxiang Li. *Resources*: Tong Tong, Xiaoyan Zhang, Yanfen Cui, and Xinxiang Li. *Software*: Jing Gong. *Supervision*: Tong Tong. *Validation*: Xiaoyan Zhang and Yanfen Cui. *Visualization*: Jing Gong and Yiqun Sun. *Writing of original draft*: TingDan Hu. *Writing review and editing*: Tong Tong and Yiqun Sun. All authors have read and approved the final manuscript.

## CONFLICT OF INTEREST STATEMENT

The authors declare no conflict of interest.

## ETHICS STATEMENT

This multicenter study was conducted in accordance with the Declaration of Helsinki. Regarding patients in the TD, external validation dataset 1 and external validation dataset 2, ethics approval was obtained from the Institutional Review Board of Fudan University Shanghai Cancer Center (Shanghai, China; Approval No.1612167−18), Peking University Cancer Hospital (Beijing, China; Approval No.2020KT53), and Shanxi Province Cancer Hospital (Shanxi, China; Approval No.202011), respectively. The retrospective nature exempted informed consent from being required. The study was authorized by the Ethics Committee of Fudan University Shanghai Cancer Center (Shanghai, China; Approval No. 1807188−10) with regard to patients in the prospective validation cohort. Informed consent was required to be obtained from each patient, and written consent was obtained from the study participants.

## Supporting information

Supporting Information

## Data Availability

Owing to patient privacy concerns, patient data are not publicly accessible. However, they are available upon reasonable request from the corresponding author (t983352@126.com).
