# Supplementary material for: Magnetic resonance imaging‐based radiomics analysis for prediction of treatment response to neoadjuvant chemoradiotherapy and clinical outcome in patients with locally advanced rectal cancer: A large multicentric and validated study
Source: MedComm (2020). 2024 Jun 20;5(7):e609. doi: 10.1002/mco2.609 (PMC11190348; doi:10.1002/mco2.609)
Supplement: Supplementary file 1 — Supporting Information [file MCO2-5-e609-s001.docx]

**Title: MRI-based radiomics analysis for prediction of treatment response to neoadjuvant chemoradiotherapy and clinical outcome in patients with locally advanced rectal cancer: A large multicentric and validated study**

**Authors:**

TingDan Hu, MD^1^*, Jing Gong, PhD ^1^*, YiQun Sun, PhD^1^*, MengLei Li, MD^1^，ChongPeng Cai, MD^1^，XinXiang Li, PhD^2^, YanFen Cui, PhD^3^†，XiaoYan Zhang, PhD^4^†，Tong Tong, PhD^1^†

**Affiliations：**

**^1^** Department of Radiology, Fudan University Shanghai Cancer Center, Department of Oncology, Shanghai Medical College, Fudan University, Shanghai, P.R China.

**^2^** Department of Colorectal Surgery, Fudan University Shanghai Cancer Center, Department of Oncology, Shanghai Medical College, Fudan University, Shanghai, P.R China.

^3^ Department of Radiology, Shanxi Province Cancer Hospital, Shanxi Hospital Affiliated to Cancer Hospital, Chinese Academy of Medical Sciences, Cancer Hospital Affiliated to Shanxi Medical University, Taiyuan, P.R China.

^4^ Key Laboratory of Carcinogenesis and Translational Research (Ministry of Education/Beijing), Department of Radiology, Peking University Cancer Hospital and Institute, Beijing, China.

* TingDan Hu, Jing Gong, and YiQun Sun contributed equally to this work

†Corresponding authors

**Corresponding authors:**

**Tong Tong, Ph.D**

No. 270, Dongan Rd, Shanghai 200032, People’s Republic of China

E-mail: [t983352@126.com](mailto:t983352@126.com)

ORCID：https://orcid.org/0000-0002-9180-8181

**XiaoYan Zhang, Ph.D**

No.52 Fu Cheng Road, Beijing 100142, Hai Dian District, China

E-mail: sunnyrose.zhang@gmail.com

**YanFen Cui, Ph.D**

No.3 Innovation Street, Xiaodian District, Taiyuan City, Shanxi Province, China

E-mail: yanfen210@126.com

**Methods and Materials**

**Evaluation criteria for MRI markers in rectal cancer**

1. mrT stage

T1-2, intact hypointense line surrounding the rectum on MRI; T3, interruption of the hypointense muscularis propria with specular or nodular extension of the tumour signal beyond the rectal wall into the mesorectal fat; T4a, invasion of the peritoneum or peritoneal reflection; T4b, invasion of other organs or structures outside the mesorectum

1. mrN stage

N0, no suspicious lymph nodes; N1, 1-3 suspicious lymph nodes/no suspicious lymph nodes but tumour deposits; N2, ≥3 suspicious lymph nodes

Suspicious if (1) short axis diameter ≥9 mm; (2) short axis diameter 5–8 mm and ≥2 morphologically suspicious characteristics; (3) short axis diameter < 5 mm and 3 morphologically suspicious characteristics; and (4) all mucinous lymph nodes (any size). Morphologically suspicious: round shape, irregular border, and heterogeneous signal

1. MRI-identified MRF involvement—the shortest distance from the outermost margin of the main tumour extension, tumour deposits, or suspicious lymph nodes to the mesorectal fascia; > 1 mm was considered negative, and ≤1 mm was considered positive
2. MRI-identified EMVI—intermediate signal intensity within vessels adjacent to the tumour, with only slight expansion of the contour and caliber of the vessels or with obvious abnormal vessel contour and/or nodular expansion of vessels indicating definite tumour invasion

**MRI acquisition protocol**

All patients underwent a conventional rectal MRI protocol that included DWI and T2WI within two weeks before nCRT. No bowel preparation (antispasmodic medication or rectal distention) was performed before the MRI examination. The axial DWIs had diffusion sensitivity values of 0 and 800 s/mm^2^ applied in tridirectional (x, y, and z) diffusion gradients using a single-shot echo-planar imaging sequence, and conventional DWIs were obtained from the DWI data based on this sequence. ADC maps were subsequently calculated in a monoexponential decay model using both b values.

**Radiomics feature extraction methodology**

In our study, a total of 1237 imaging features for each patient were extracted, of which 1130 features were from the T2W images and 107 were from the ADC images. All radiomics features were calculated automatically with PyRadiomics software. The 1130 features extracted from T2W could be divided into four categories: (a) 18 first-order statistical features; (b) 14 shape-based 3D features; (c) 75 statistics-based textural features; and (d) 1023 wavelet and Laplacian of Gaussian (LoG) filtration features. The 107 features extracted from ADC images could be divided into three categories: (a) 18 first-order statistical features; (b) 14 shape-based 3D features; and (c) 75 statistics-based textural features. Detailed information on these features is available in the documentation for PyRadiomics (<http://PyRadiomics.readthedocs.io/en/latest/>).

**(1) First-order statistical features**

First-order statistics describe the distribution of voxel intensities within the image region defined by the mask through commonly used and basic metrics. A total of 18 first-order statistical features were extracted.

**(2) Shape-based 3D features**

In this group of features, we included descriptors of the three-dimensional size and shape of the region of interest (ROI). These features are independent of the grey-level intensity distribution in the ROI and are therefore only calculated on the nonderived image and mask. We extracted 14 shape-based features in our present study.

**(3) Statistics-based textural features**

Statistics-based textural features can reflect the homogeneity phenomenon of the images and the arrangement of the properties that change slowly or periodically on the body surface. The textural features extracted in our study included five types of matrix features, including 24 grey-level co-occurrence matrix (GLCM) features, 16 grey-level run length matrix (GLRLM) features, 16 grey-level size zone matrix (GLSZM) features, 5 neighbouring grey tone difference matrix (NGTDM) features, and 14 grey-level dependence matrix (GLDM) features. Determining the texture matrix representations requires the voxel intensity values within the volume of interest (VOI) to be discretized. Voxel intensities were therefore resampled into equally spaced bins using a bin width of 5 grey levels. This discretization step not only reduces the image noise but also normalizes the intensities across all patients, allowing for a direct comparison of all the calculated textural features between patients.

A GLCM classifier is used to describe the distance and angle of each pixel and calculate the correlation between two grey levels with certain directions and distances. The GLCM can reflect integrated information regarding the direction, interval, amplitude, and frequency of images. For GLRLM, the run length metrics quantify the grey-level runs in an image. A grey-level run is defined as the length of the number of pixels and of consecutive pixels that have the same grey-level value. The GLSZM describes the amount of homogeneous connected areas within the tumour volume of a certain size and intensity, thus reflecting tumour heterogeneity at the regional scale.

**(4) Wavelet and LoG filtration features**

Two image filters, wavelet and LoG, were applied to the original image to yield a corresponding derived image. An LoG spatial bandpass filter was used to derive image features at different spatial scales by tuning the filter parameters to 1.0, 3.0 and 5.0.

Wavelet transformation effectively decouples textural information by decomposing the original image into low- and high-frequency images. A discrete, one-level and undecimated three-dimensional wavelet transformation was applied to each MR image, which decomposed the original image into 8 decompositions. Considering L and H to be low-pass and high-pass functions, respectively, X is the decomposing image, and the wavelet decompositions of X are labelled XLLL, XLLH, XLHL, XLHH, XHLL, XHLH, XHHL, and XHHH. Then, eight new images that are decomposed in three directions (x, y, z) could be obtained. Since the applied wavelet decomposition was undecimated, the size of each decomposition was equal to that of the original image, and each decomposition was shift invariant. Thus, the original tumour delineation of the tumour volume could be applied directly to the decompositions after wavelet transformation. For each decomposition and LoG filtration, we computed the first-order statistical features and the statistics-based textural features; therefore, (18 + 75)*11=1023 wavelet and LoG filtration features were obtained.

**Boruta algorithm**

Boruta is a wrapper algorithm for all-relevant feature selection, where relevant features are searched in a top-down manner by comparing the importance of the original features with the importance achieved by artificially added random features. A random forest algorithm was used in each iteration to evaluate the classification and measure the feature importance, where irrelevant features were progressively eliminated. To obtain statistically significant results, the algorithm repeatedly calculated all possible subsets of the features and finally selected the minimal set of the most relevant features for optimal classification. Compared to selecting univariately predictive and nonredundant features, using Boruta could improve the generalizability and interpretability of selected features. The R package Boruta was used to build the model.

**Supplementary Figure 1**
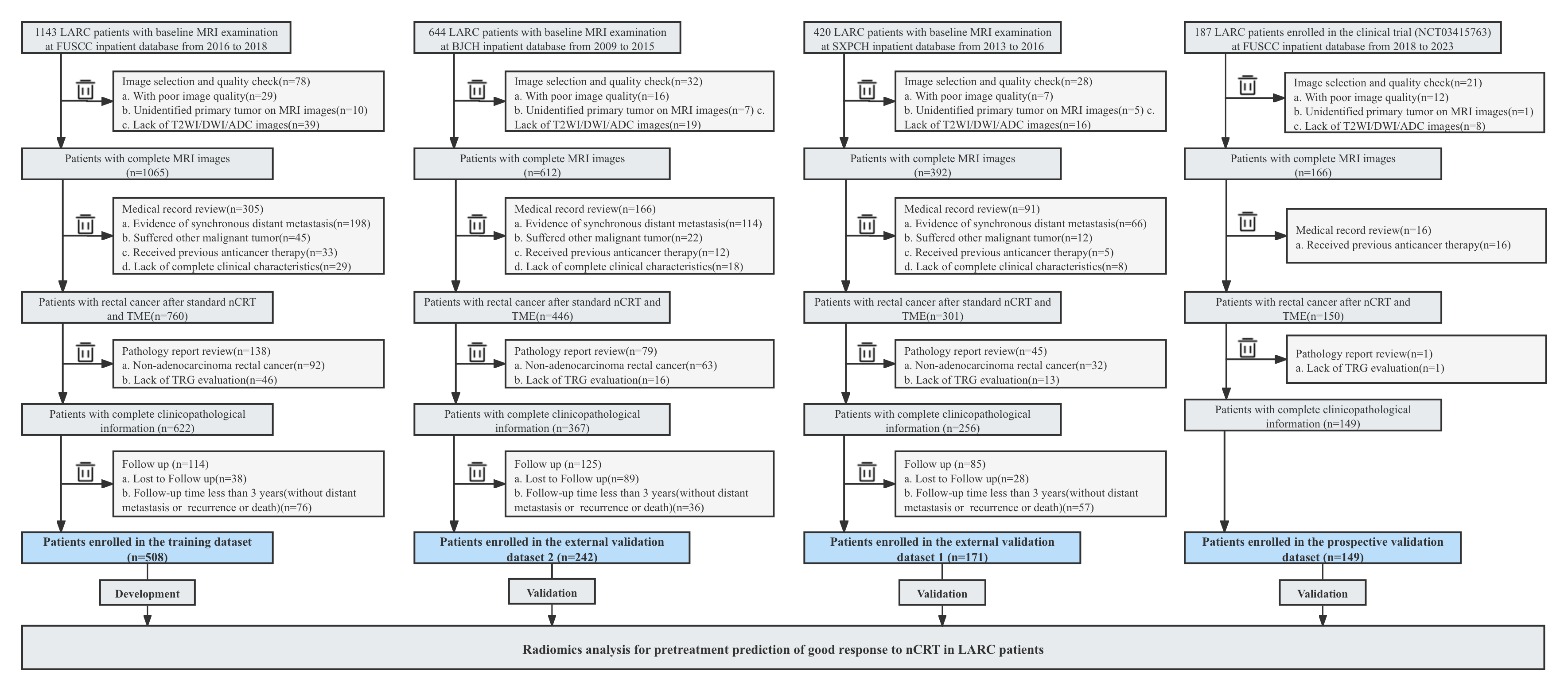


**Supplementary Figure 1**. Patients recruitment pathway.

**Supplementary Figure 2**


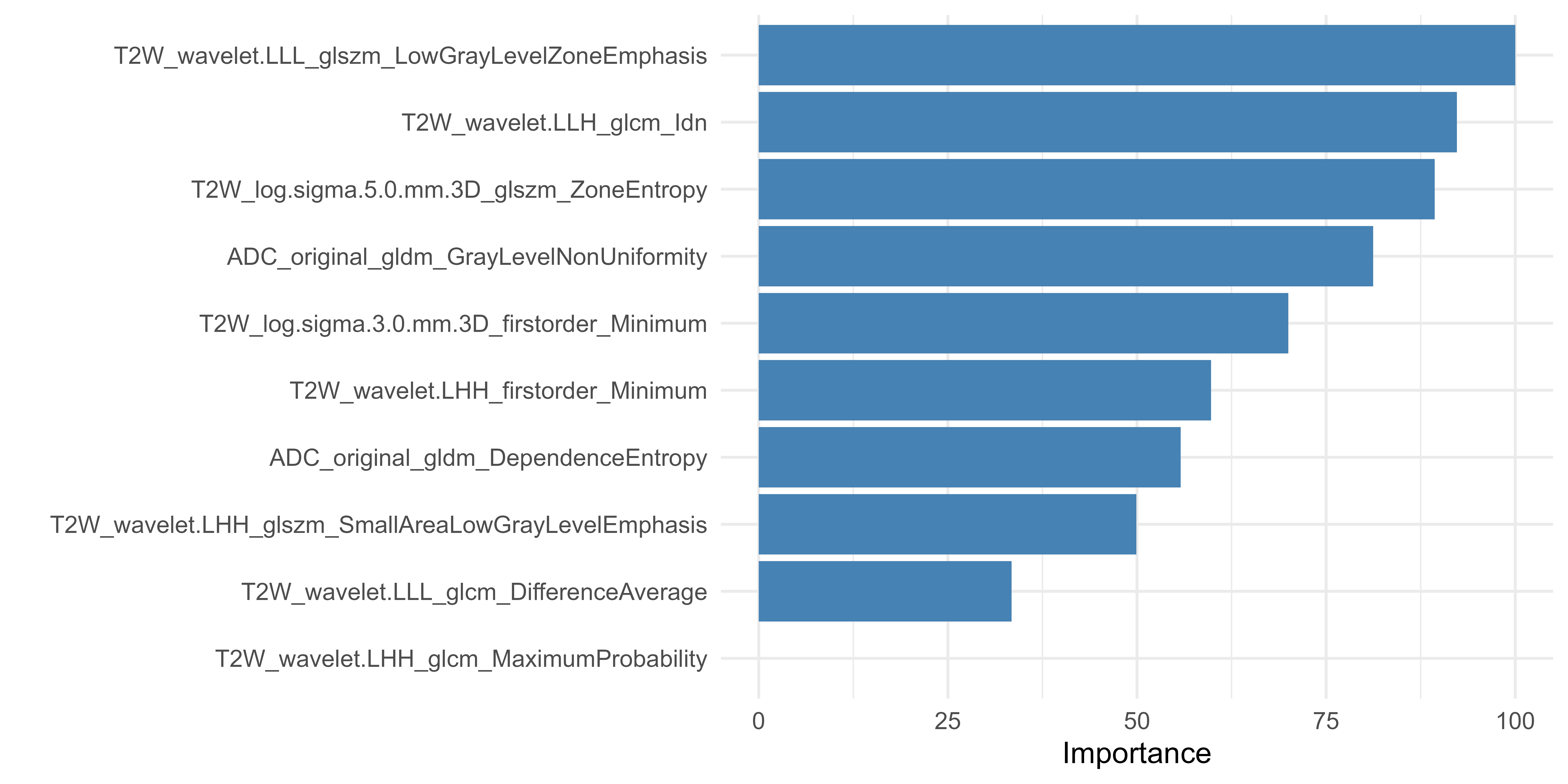


**Supplementary Figure 2**. The importance of the selected 10 features with the most predictive value, consisting of 2 features extracted from ADC images and 8 features extracted from T2W images.

**Supplementary Figure 3**


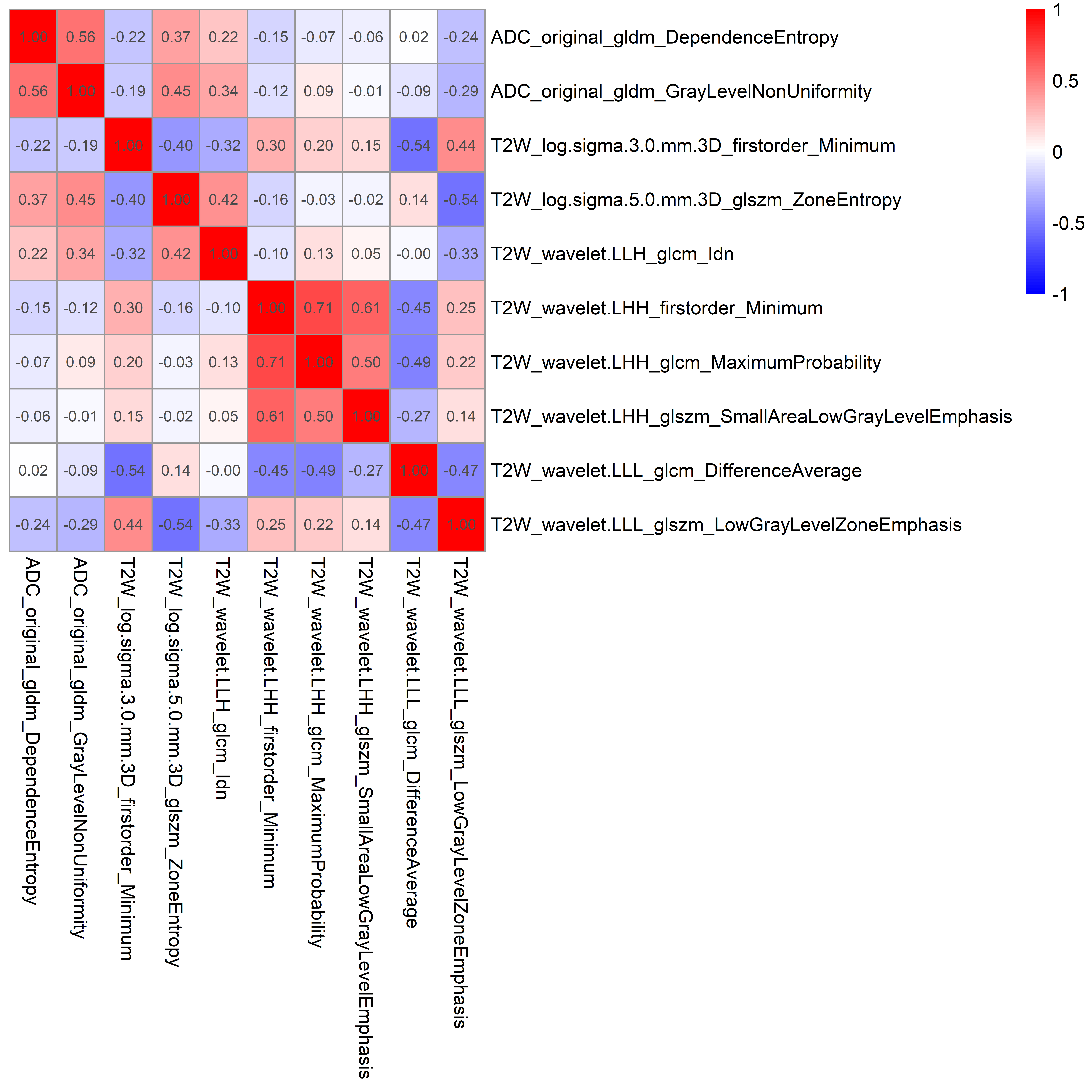


**Supplementary Figure 3**. The correlation matrix of the selected features.

**Supplementary Figure 4**

**
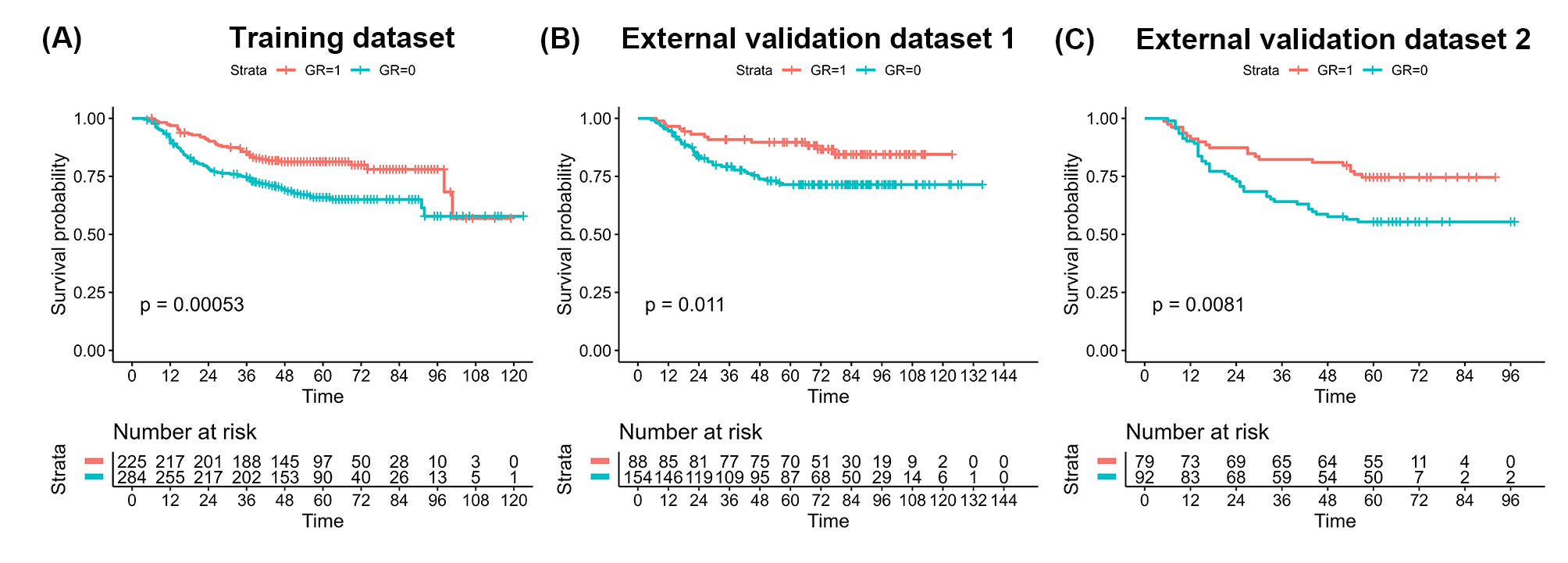
Supplementary Figure 4.** Kaplan‒Meier curves of disease-free survival between the real good-response and poor-response groups.

**
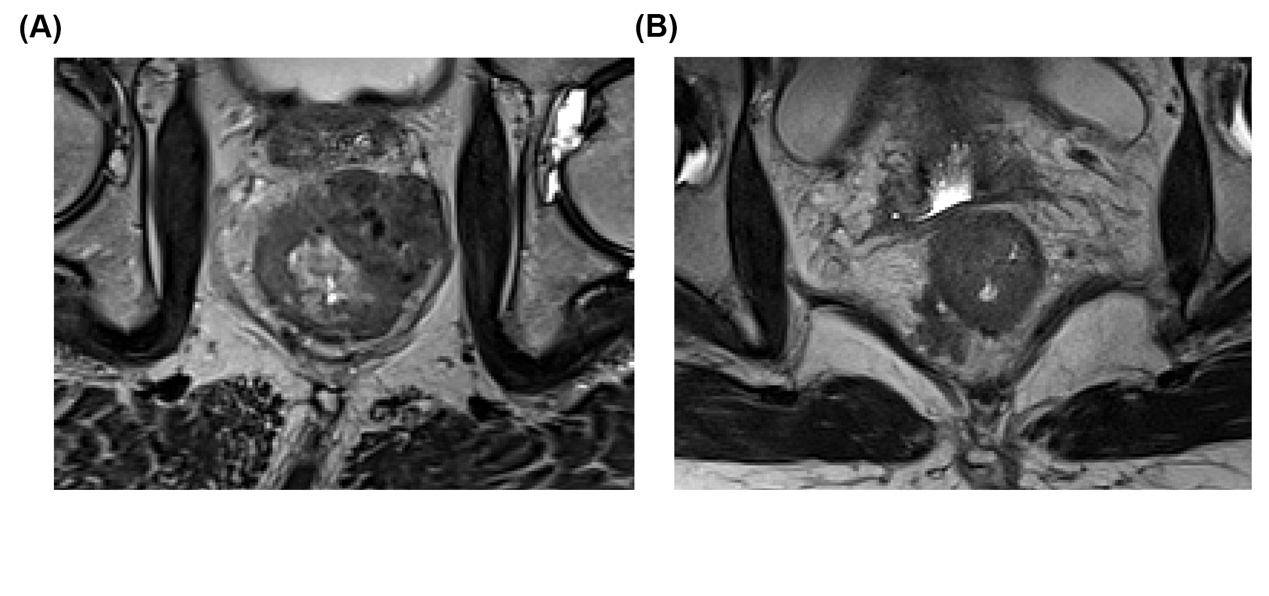
Supplementary Figure 5**

**Supplementary Figure 5**. Typical images of MRI risk factors

A) mesorectal fascia (MRF); B) extramural vascular invasion (EMVI).

| **Supplementary Table 1: Clinicopathological Characteristics of Patients with LARC in the Four Datasets** | | | | | |
| --- | --- | --- | --- | --- | --- |
| **Variables** | **Total (n = 1070)** | **TD (n = 508)** | **EVD1 (n = 242)** | **EVD2 (n = 171)** | **PVD (n = 149)** |
| Treatment response |  |  |  |  |  |
| PR | 594 (55.5%) | 284 (55.9%) | 154 (63.6%) | 92 (53.8%) | 64 (43.0%) |
| GR | 476 (44.5%) | 224 (44.1%) | 88 (36.4%) | 79 (46.2%) | 85 (57.0%) |
| Age (y) | 55.1 (44.2-66.0) | 54.8 (44.0-65.6) | 55.0 (43.8-66.2) | 54.4 (44.2-64.6) | 57.2 (45.8-68.6) |
| Sex |  |  |  |  |  |
| Female | 359 (33.6%) | 156 (30.7%) | 94 (38.8%) | 64 (37.4%) | 45 (30.2%) |
| Male | 711 (66.4%) | 352 (69.3%) | 148 (61.2%) | 107 (62.6%) | 104 (69.8%) |
| CEA level |  |  |  |  |  |
| ≤5ng/ml | 797 (74.5%) | 399 (78.5%) | 175 (72.3%) | 123 (71.9%) | 100 (67.1%) |
| >5ng/ml | 273 (25.5%) | 109 (21.5%) | 67 (27.7%) | 48 (28.1%) | 49 (32.9%) |
| Distance (cm) | 5.2 (3.0-7.3) | 5.3 (3.2-7.3) | 5.2 (2.9-7.4) | 5.5 (3.0-7.9) | 4.8 (2.9-6.6) |
| Tumour size (cm) | 5.2 (3.7-6.7) | 5.3 (3.6-7.0) | 5.5 (4.1-6.8) | 4.8 (3.5-6.1) | 5.1 (4.0-6.2) |
| mrT stage |  |  |  |  |  |
| T2 | 66 (6.2%) | 19 (3.7%) | 30 (12.4%) | 11 (6.4%) | 6 (4.0%) |
| T3 | 844 (78.9%) | 409 (80.5%) | 180 (74.4%) | 131 (76.6%) | 124 (83.2%) |
| T4 | 160 (15.0%) | 80 (15.8%) | 32 (13.2%) | 29 (17.0%) | 19 (12.8%) |
| mrN stage |  |  |  |  |  |
| N0 | 81 (7.6%) | 39 (7.7%) | 14 (5.8%) | 18 (10.5%) | 10 (6.7%) |
| N1 | 361 (33.7%) | 161 (31.7%) | 84 (34.7%) | 61 (35.7%) | 55 (36.9%) |
| N2 | 628 (58.7%) | 308 (60.6%) | 144 (59.5%) | 92 (53.8%) | 84 (56.4%) |
| mrMRF |  |  |  |  |  |
| negative | 687 (64.2%) | 328 (64.6%) | 152 (62.8%) | 108 (63.2%) | 99 (66.4%) |
| positive | 383 (35.8%) | 180 (35.4%) | 90 (37.2%) | 63 (36.8%) | 50 (33.6%) |
| mrEMVI |  |  |  |  |  |
| negative | 635 (59.3%) | 311 (61.2%) | 116 (47.9%) | 117 (68.4%) | 91 (61.1%) |
| positive | 435 (40.7%) | 197 (38.8%) | 126 (52.1%) | 54 (31.6%) | 58 (38.9%) |
| Metastasis/Recurrence |  |  |  |  |  |
| 0 | 700 (72.7%) | 370 (72.8%) | 189 (78.1%) | 110 (64.3%) | / |
| 1 | 263 (27.3%) | 138 (27.2%) | 53 (21.9%) | 61 (35.7%) | / |
| Time.DFS | 54.2 (26.7-81.6) | 51.1 (26.1-76.1) | 66.7 (34.4-99.0) | 50.3 (27.8-72.8) | / |
| *Abbreviations: TD, training dataset; EVD1, external validation dataset 1; EVD2, external validation dataset 2; PVD, prospective validation dataset; PR, poor response; GR, good response; CEA, carcinoembryonic antigen; MRF, mesorectal fascia; EMVI, extramural venous invasion; DFS, disease-free survival.* | | | | | |
| **P < 0.05.* |  |  |  |  |  |

| **Supplementary Table 2. radiomics feature with ICC<0.75** | |
| --- | --- |
| **Sequences** | **Features** |
| ADC | original_glcm_Autocorrelation |
|  | original_glcm_SumSquares |
|  | original_glrlm_RunLengthNonUniformityNormalized |
|  | original_glrlm_RunPercentage |
|  | original_glszm_LargeAreaEmphasis |
|  | original_glszm_LargeAreaHighGrayLevelEmphasis |
|  | original_glszm_LargeAreaLowGrayLevelEmphasis |
|  | original_gldm_LargeDependenceHighGrayLevelEmphasis |
|  | original_gldm_LowGrayLevelEmphasis |
|  | original_gldm_SmallDependenceEmphasis |
|  | original_ngtdm_Coarseness |
| T2WI | original_firstorder_Kurtosis |
|  | original_firstorder_Variance |
|  | original_glcm_ClusterProminence |
|  | original_glcm_ClusterShade |
|  | original_glcm_ClusterTendency |
|  | original_glcm_SumSquares |
|  | original_glrlm_GrayLevelVariance |
|  | original_gldm_GrayLevelVariance |
|  | log.sigma.3.0.mm.3D_glcm_ClusterProminence |
|  | log.sigma.3.0.mm.3D_glcm_ClusterShade |
|  | log.sigma.5.0.mm.3D_glcm_ClusterProminence |
|  | log.sigma.5.0.mm.3D_glcm_ClusterShade |
|  | wavelet.LLH_glcm_ClusterShade |
|  | wavelet.LLL_firstorder_Kurtosis |
|  | wavelet.LLL_firstorder_Variance |
|  | wavelet.LLL_glcm_ClusterProminence |
|  | wavelet.LLL_glcm_ClusterShade |
|  | wavelet.LLL_glcm_ClusterTendency |
|  | wavelet.LLL_glcm_SumSquares |
|  | wavelet.LLL_glrlm_GrayLevelVariance |
|  | wavelet.LLL_glszm_GrayLevelVariance |
|  | wavelet.LLL_gldm_GrayLevelVariance |
|  | lbp.3D.m1_firstorder_Maximum |
|  | lbp.3D.m1_firstorder_Minimum |
|  | lbp.3D.m1_firstorder_Range |
|  | lbp.3D.m2_firstorder_Minimum |

| **Supplementary Table 3. The Radiomics Quality Score (RQS)** | | | | |
| --- | --- | --- | --- | --- |
| Criteria | | Points | Methods | Score |
| 1 | Image protocol quality - well-documented image protocols (for example, contrast, slice thickness, energy, etc.) and/or usage of public image protocols allow reproducibility/replicability | + 1 (if protocols are well-documented) | Imaging protocol is documented. | 1 |
|  |  | + 1 (if public protocol is used) |  |  |
| 2 | Multiple segmentations - possible actions are: segmentation by different physicians/algorithms/software, perturbing segmentations by (random) noise, segmentation at different breathing cycles. Analyse feature robustness to segmentation variabilities | + 1 | Image segmentation was firstly performed by by a colorectal MRI radiologists (Reader 1), and a total of 50 patients were randomly selected, their MRI images were dependently delineated again one month later by reader 1 and another senior radiologist to assess the  inter- and intra-observer reproducibility | 1 |
| 3 | Phantom study on all scanners - detect inter-scanner differences and vendor-dependent features. Analyse feature robustness to these sources of variability | + 1 | 0 | 0 |
| 4 | Imaging at multiple time points - collect images of individuals at additional time points. Analyse feature robustness to temporal variabilities (for example, organ movement, organ expansion/ shrinkage) | + 1 | 0 | 0 |
| 5 | Feature reduction or adjustment for multiple testing - decreases the risk of overfitting. Overfitting is inevitable if the number of features exceeds the number of samples. Consider feature robustness when selecting features | - 3 (if neither measure is implemented) | The Mann‒Whitney U test, Spearman’s correlation analysis and Boruta feature selection was gradually performed for selecting robust radiomics features with intraclass correlation coefficient (ICC) ≥ 0.75. | 3 |
|  |  | + 3 (if either measure is implemented) |  |  |
| 6 | Multivariable analysis with non radiomics features (for example, EGFR mutation) - is expected to provide a more holistic model. Permits correlating/inferencing between radiomics and non radiomics features | + 1 | The radiomics signature generated by the best classifier was defined as a new feature set and further used to construct a combined model with the joint of independent clinical-MRI features. | 1 |
| 7 | Detect and discuss biological correlates - demonstration of phenotypic differences (possibly associated with underlying gene–protein expression patterns) deepens understanding of radiomics and biology | + 1 | 0 | 0 |
| 8 | Cut-off analyses - determine risk groups by either the median, a previously published cut-off or report a continuous risk variable. Reduces the risk of reporting overly optimistic results | + 1 | The receiver operating characteristic (ROC) curves were plotted and the best cut-off point was obtained by maximizing the Youden index in the training dataset and applied in three validation datasets to reduce the risk of overfitting and reporting overly optimistic results. | 1 |
| 9 | Discrimination statistics - report discrimination statistics (for example, C-statistic, ROC curve, AUC) and their statistical significance (for example, p-values, confidence intervals). One can also apply resampling method (for example, bootstrapping, cross-validation) | + 1 (if a discrimination statistic and its statistical significance are reported) | The area under the ROC curve (AUC) was calculated, the sensitivity, specificity and predictive accuracy were measured. The 95% confidence interval (CI) of the AUC was determined by the bootstrap resampling method with 1000 replicates. | 2 |
|  |  | + 1 (if a resampling method technique is also applied) |  |  |
| 10 | Calibration statistics - report calibration statistics (for example, Calibration-in-the-large/slope, calibration plots) and their statistical significance (for example, P-values, confidence intervals). One can also apply resampling method (for example, bootstrapping, cross-validation) | + 1 (if a calibration statistic and its statistical significance are reported) | 0 | 0 |
|  |  | + 1 (if a resampling method technique is also applied) |  |  |
| 11 | Prospective study registered in a trial database - provides the highest level of evidence supporting the clinical validity and usefulness of the radiomics biomarker | + 7 (for prospective validation of a radiomics signature in an appropriate trial) | Our study included a prospective validation dataset recruited from the clinical trail (Registration No. NCT03415763) in Fudan University Shanghai Cancer Center to validate the model performance. | 7 |
| 12 | Validation - the validation is performed without retraining and without adaptation of the cut-off value, provides crucial information with regard to credible clinical performance | - 5 (if validation is missing) | Our study included three dependent datasets, including two external validation datasets and a prospective validation dataset, to validate the model performance. | 5 |
|  |  | + 2 (if validation is based on a dataset from the same institute) |  |  |
|  |  | + 3 (if validation is based on a dataset from another institute) |  |  |
|  |  | + 4 (if validation is based on two datasets from two distinct institutes) |  |  |
|  |  | + 4 (if the study validates a previously published signature) |  |  |
|  |  | + 5 (if validation is based on three or more datasets from distinct institutes) |  |  |
| 13 | Comparison to ‘gold standard’ - assess the extent to which the model agrees with/is superior to the current ‘gold standard’ method (for example, TNM-staging for survival prediction). This comparison shows the added value of radiomics | + 2 | 0 | 0 |
| 14 | Potential clinical utility - report on the current and potential application of the model in a clinical setting (for example, decision curve analysis). | + 2 | Decision curve analysis (DCA) was performed to estimate the clinical usefulness of the models at different threshold probabilities. | 2 |
| 15 | Cost-effectiveness analysis - report on the cost-effectiveness of the clinical application (for example, QALYs generated) | + 1 | 0 | 0 |
| 16 | Open science and data - make code and data publicly available. Open science facilitates knowledge transfer and reproducibility of the study | + 1 (if scans are open source) | The scans are open source. the region of interest segmentation is performed on the open source (ITK-SNAP). The radiomics features are defined and publically available via pyradiomics. The code will be made available upon acceptance of the paper. | 4 |
|  |  | + 1 (if region of interest segmentations are open source) |  |  |
|  |  | + 1 (if code is open source) |  |  |
|  |  | + 1 (if radiomics features are calculated on a set of representative ROIs and the calculated features and representative ROIs are open source) |  |  |
| Total score of this study | | | | 27/36 |

| **Supplementary Table 4: The detailed MRI parameters at the three hospitals** | | | | | | | | | |
| --- | --- | --- | --- | --- | --- | --- | --- | --- | --- |
| **Hospital** | **Scanner(manufactural models name)** | **Sequence** | **TR/TE(ms)** | **FOV(mm)** | **Matrix** | **Slice Thickness (mm)** | **Slice Gap(mm)** | **Slices** | **Flip Angle** |
| Fudan University Shanghai Cancer Center | Siemens 3.0T(skyra) | T2WI | 1500/100 | 230 | 320x291 | 1.5 | 2 | 64 | 135 |
|  |  | DWI | 4900/60 | 200 | 140x126 | 5.5 | 5.5 | 20 | 180 |
|  | GE 3.0T(signa HDxt) | T2WI | 3800/105 | 200 | 384x224 | 3 | 4 | 22 | 90 |
|  |  | DWI | 2800/66 | 300 | 128x128 | 6 | 8 | 28 | 90 |
|  | Siemens 3.0T(verio) | T2WI | 1500/98 | 230 | 320x291 | 1.5 | 2 | 64 | 135 |
|  |  | DWI | 4500/81 | 200 | 140x124 | 5 | 5 | 20 | 90 |
|  | Siemens 3.0T(Prisma) | T2WI | 4290/89 | 100 | 320x256 | 3.5 | 3.5 | 21 | 160 |
|  |  | DWI | 4500/46 | 75 | 120x90 | 5.5 | 5.5 | 20 | 90 |
|  | GE 3.0T(SIGNA pioneer) | T2WI | 1428/83 | 100 | 320x256 | 3.3 | 3.3 | 24 | 111 |
|  |  | DWI | 5602/62 | 50 | 140x70 | 3.3 | 3.3 | 24 | 90 |
| Beijing Cancer Hospital | GE 3.0T | T2WI | 5550/110 | 180 | 352x320 | 3 | 3.3 | 40 | 90 |
|  | (DISCOVERY MR750) | DWI | 2800/66 | 340 | 128x128 | 4 | 4.0 | 24 | 90 |
| Shanxi Cancer Hospital | Philips 3.0T(Achieva) | T2WI | 3000/80 | 180 | 330x223 | 3 | 3.3 | 20 | 90 |
|  |  | DWI | 2750/53.4 | 375 | 187x124 | 5 | 5.5 | 32 | 90 |

*Abbreviations: TR, repetition time; TE, echo time; FOV, field of view; T2WI, T2 weighted imaging; DWI, diffusion weighted imaging.*
